# Lymphatic Invasion is an Independent Adverse Prognostic Factor in Patients with Colorectal Liver Metastasis

**DOI:** 10.1245/s10434-015-4562-8

**Published:** 2015-05-19

**Authors:** Jannemarie A. M. de Ridder, Nikki Knijn, Bastiaan Wiering, Johannes H. W. de Wilt, Iris D. Nagtegaal

**Affiliations:** Department of Surgical Oncology, Radboud University Medical Center, Nijmegen, The Netherlands; Department of Pathology, Radboud University Medical Center, Nijmegen, The Netherlands; Department of Surgery, Slingeland Hospital, Doetinchem, The Netherlands

## Abstract

**Background:**

For a selection of patients with colorectal liver metastases (CRLM), liver resection is a curative option. In order to predict long-term survival, clinicopathologic risk scores have been developed, but little is known about histologic factors and their prognostic value for disease-free and overall survival. The objective of the present study was to assess possible prognostic histologic factors in patients with solitary CRLM treated with liver resection who did not receive neoadjuvant treatment.

**Methods:**

Patients with solitary CRLM who underwent liver resection between 1992 and 2011 were evaluated for clinical prognostic factors. Histologic analyses on tumor thickness at the tumor–normal interface, presence of a fibrotic capsule, intrahepatic vascular invasion, lymphatic invasion, or bile duct invasion and perineural growth were performed, using immunohistochemistry.

**Results:**

A total of 124 patients were analyzed with a median follow-up of 41 months (range 1–232 months). There was no association between histologic factors and disease-free survival in multivariate analysis. In multivariate analysis, intrahepatic lymphatic invasion was associated with a decreased overall survival (41.9 vs. 61.0 months; *p* = 0.041), especially in combination with vascular invasion (*n* = 15) (28.1 vs. 62.2 months; *p* < 0.0001). In addition, size over 50 mm (29.2 vs. 65.9 months; *p* = 0.004) and interval less than 12 months between resection of the primary tumor and diagnosis of liver metastasis (49.0 vs. 91.5 months: *p* = 0.019) were also independent adverse prognostic factors.

**Conclusions:**

Intrahepatic lymphatic invasion, especially in combination with vascular invasion, is an important adverse prognostic factor for overall survival in patients with solitary CRLM after liver resection.

**Electronic supplementary material:**

The online version of this article (doi:10.1245/s10434-015-4562-8) contains supplementary material, which is available to authorized users.

Colorectal cancer is one of the leading causes of cancer death worldwide as a result of its considerable risk of development of metastases.^1^ When metastatic disease is confined to the liver, partial liver resection is the only curative therapeutic option, with 5-year overall survival (OS) percentages between 20 and 60 %, depending on patient and tumor characteristics.^2^–^4^ In order to explain these varying survival rates, different clinicopathologic risk scores have been developed. In many of these risk scores, nodal status of the primary tumor, size and number of the colorectal liver metastases (CRLM), disease-free interval from treatment of the primary until detection of the CRLM, and preoperative level of carcinoembryonic antigen (CEA) are combined to predict long-term survival.^5^–^9^ These scoring systems are relevant with respect to prediction of survival, but to our knowledge, they have not been used for risk stratification in controversial areas such as the administration of neoadjuvant or adjuvant systemic therapy or surveillance.

In primary colorectal cancer histologic factors such as extramural venous invasion, perineural growth, lymphatic invasion, angioinvasion, and diffuse growth pattern have been associated with poorer survival outcomes.^10^,^11^ Extramural venous invasion in particular is considered a poor prognostic factor, and as a result, patients with extramural venous invasion in stage II colon cancer are considered candidates for adjuvant systemic treatment.^12^ Very little is known about the impact of histologic features of colorectal liver metastases on OS, as described in a recent review.^13^

Vascular invasion, bile duct invasion, or lymphatic invasion by tumor cells in CRLM have all been suggested as prognostic factors for long-term survival.^5^,^14^–^23^ Perineural growth, the presence of a fibrous capsule, and tumor thickness at the tumor–normal interface have also been linked to survival in patients with CRLM.^14^,^15^,^19^,^24^–^26^ Variations in definitions and selection of patients have limited the impact of these studies. Furthermore, none of these previous studies has evaluated multiple histologic factors of the liver resection specimens, in combination with established risk scores in a homogenous group of patients. Most studies included patients who underwent neoadjuvant therapy as well as chemotherapy-naive patients, patients with multiple liver metastases, or patients with extrahepatic disease.^5^,^14^–^21^,^23^,^24^ The results of these previous studies might be biased because of the known changes in histologic features observed in liver metastases after systemic therapy, and the possible heterogeneous nature of multiple metastases.^27^–^30^

The objective of the current study was to assess possible prognostic histologic factors for long-term survival in patients with solitary colorectal liver metastases who underwent a complete (R0) liver without neoadjuvant systemic therapy.

## Materials and Methods

### Patients

Patients were identified who underwent complete (R0) liver resection for a solitary CRLM between 1992 and 2011 in a tertiary referral hospital. R0 resections were defined as liver resections with clear resection margins in patients who did not have evidence of disease in any other locations. Demographics and clinicopathologic factors with regard to the primary tumor, as well as the liver metastasis, were collected per patient. Special attention was given to the four different items from the clinical risk score according to Fong et al.: nodal status of the primary tumor; preoperative CEA level and size of the metastasis, and interval between resection of the primary tumor and diagnosis of CRLM.^9^ It is unknown whether systemic treatment influences the presence of certain histopathology factors and therefore patients who were treated with neoadjuvant systemic therapy were excluded from the current study. Patients who died from postoperative complications, defined as within 30 days after liver resection, were also excluded. Patients underwent follow-up according to our current Dutch follow-up guidelines, with regular outpatient visits, CEA testing and computed tomographic scans of chest and abdomen.

### Histopathology

R0 liver resection specimens with a solitary CRLM were selected from the archive. Routine workup consisted of sampling of macroscopically normal liver tissue, invasive front of the metastasis, and additional tumor blocks, depending on the size of metastasis. Slide revision was performed independently by two investigators (JdR, NK). Discrepancies were resolved by simultaneous reexamination of the slides by both investigators using a two-headed microscope. In case of discrepancy, the senior pathologist (IN) made the final call.

**Fig. 1:**
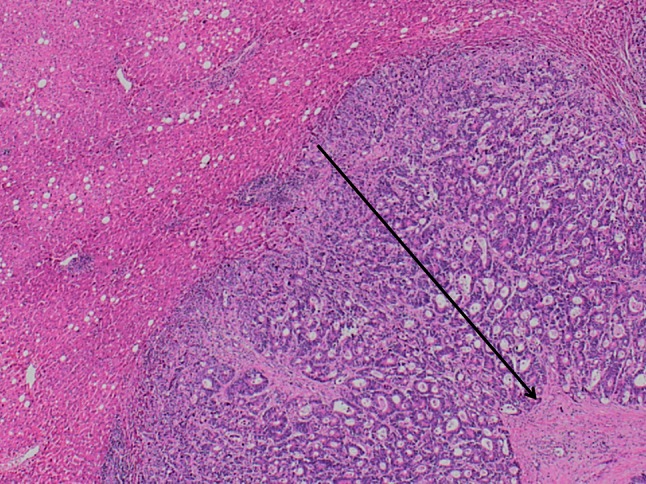
Tumor thickness at tumor–normal interface; *arrow* indicates correct measurement with uninterrupted layer of tumor cells. Original magnification, ×10

Tumor thickness at the tumor–normal interface was determined in routine slides. Tumor–normal interface was defined as the interface between tumor and normal liver tissue, as described by Maru et al. and validated by others.^26^,^31^,^32^ In all tumors, tumor thickness was measured with a ruler at multiple foci, and maximum tumor thickness was used and defined as uninterrupted layers of tumor cells without admixed fibrotic stroma, acellular mucin, or nonneoplastic liver parenchyma. The median tumor thickness at tumor–normal interface was used to divide the patient group in a group with a larger and a smaller layer of vital tumor cells (Fig. 1).

**Fig. 2:**
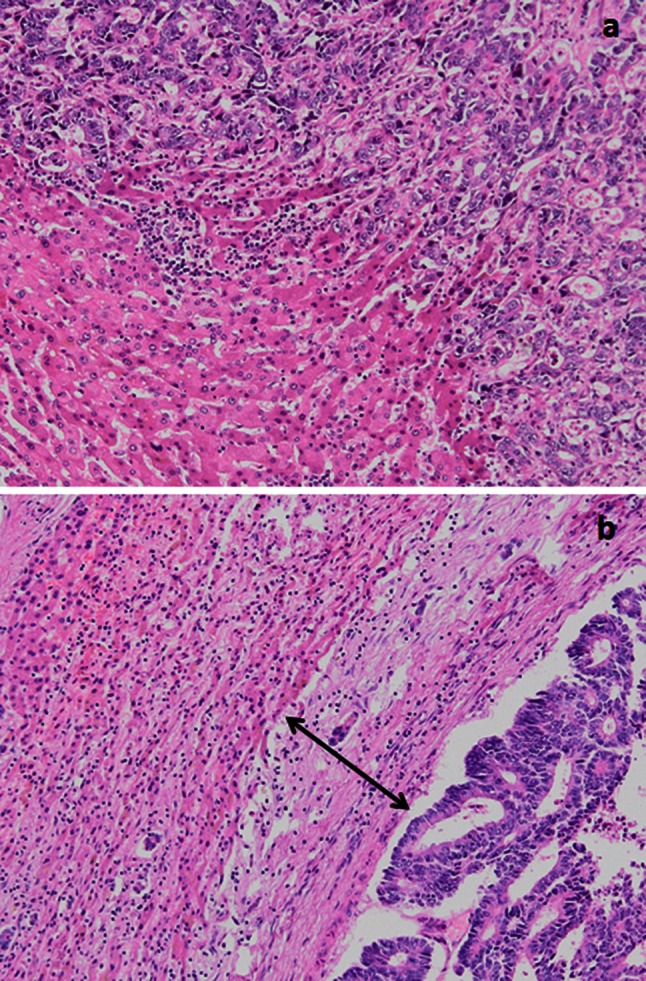
**a** Colorectal liver metastasis without fibrous capsule. Original magnification, ×20. **b** Colorectal liver metastasis with fibrous capsule (*arrow*). Original magnification, ×20

The presence of a fibrotic capsule around the metastasis was evaluated in routine slides. The fibrous tissue between tumors and liver parenchyma was classified as absent (no fibrous tissue observed) or present: tumor was separated from the liver parenchyma by several layers of collagen bundles in histologic sections (Fig. 2).

### Immunohistochemistry and Scoring Methods

Immunohistochemistry was performed as previously described.^33^ Antibodies, clones, dilution, and retrieval methods are summarized in Supplementary Table 1.

**Fig. 3:**
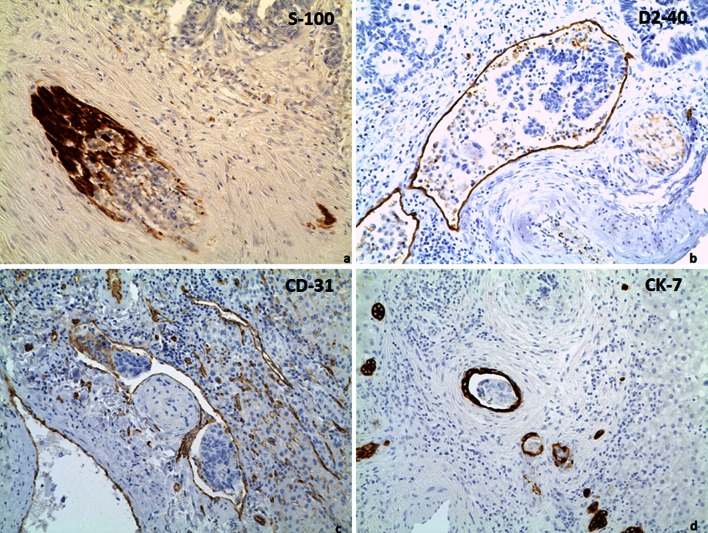
Different forms of intrahepatic invasion by tumor cells. **a** Perineural growth showing S-100 reactivity. **b** Lymphatic invasion showing D2-40 reactivity. **c** Vascular invasion showing CD-31 reactivity. **d** Bile duct invasion showing CK-7 reactivity. Original magnification, ×20

Perineural growth was defined as a nerve, identified by S-100 staining, being surrounded by tumor cells for at least three quarters of the circumference and was scored as being present or absent (Fig. 3a).

Lymphatic invasion was defined as single tumor cells or cell clusters visible within vessels that showed immunoreactivity for D2-40 but not for CD31. Lymphatic invasion was scored as being present or absent (Fig. 3b).

Vascular invasion was defined as single tumor cells or cell clusters visible within vessels that showed immunoreactivity for CD31 but not for D2-40. It was scored as being present or absent (Fig. 3c).

Bile duct invasion was defined as single tumor cells or cell clusters (CK7 negative) visible within bile ducts that showed immunoreactivity for CK7. It was also scored as being present or absent (Fig. 3d).

### Outcome

Primary outcomes were disease-free survival (DFS) and OS. DFS was defined as the interval in months between liver resection and disease recurrence, death, or last follow-up. OS was defined as the interval in months between liver resection and death or date of last follow-up.

### Statistical Analysis

Pearson’s Chi square test was used to calculate correlations between the various histologic features. Survival curves were estimated by the Kaplan–Meier method and compared by log rank testing. Multivariate analysis was performed using Cox proportional hazard model, and variables were included that were associated with survival in univariate analysis with a *p* value of <0.10. SPSS statistical software, version 18.0 (IBM, Armonk, NY, USA) was used for all statistical analysis. A *p* value of <0.05 was considered statistically significant.

## Results

### Patients

Between January 1992 and March 2011, a total of 383 patients underwent liver resection for metastatic disease. After excluding patients with multiple metastases, 135 patients remained who were surgically treated (R0) for solitary CRLM. Eleven patients were excluded because they received neoadjuvant chemotherapy (*n* = 5), were lost to follow-up (*n* = 2), or died within 30 days after liver resection (*n* = 4). A total of 124 patients were eligible to be included in the current study, 76 men (61.3 %) and 48 women (38.7 %). Median age at time of resection was 64 years (range 40–80 years). Liver metastasis were detected at a median of 8.8 months (range 0–82 months) after resection of the primary tumor. Median size of the metastasis was 35 mm (range 10–130 mm). Median follow-up was 41 months (range 1–232 months). In the complete study population, median DFS was 28 months (range 1–228 months) with a median OS of 57 months (range 1–232 months) and a 5-year survival of 48.1 %.

### Histopathologic Tumor Features

#### Fibrous Capsule and Tumor Thickness

In 34.4 % of patients (*n* = 43), the liver metastasis was surrounded by a fibrous capsule. Presence of a fibrous capsule was not associated with DFS, but it was associated with an improved OS of 109.3 months, versus 56.7 months in patients without a fibrous capsule (*p* = 0.037). In multivariate analysis, presence of a fibrous capsule was not an independent risk factor for OS (Tables 1, 2).

**Table 1:** Relation of clinical and histologic factors with DFS after liver resection in patients with solitary CRLM

	*n*	%	Median DFS	UV *p* value	MV *p* value
Size (mm)					
≤50	93	75	50.1	0.002*	0.020*
>50	31	25	14.5		
CEA (ng/ml)					
≤200	121	97.6	27.5	0.508	–
>200	3	2.4	40.6		
DFI (months)					
≤12	72	58.1	27.8	0.232	–
>12	52	41.9	25.4		
Nodal state primary					
N0	54	43.5	35.7	0.446	
N+	70	56.5	27.5		–
Adjuvant therapy					
No	106	85.5	20.2	0.013*	0.025*
Yes	18	14.5	>50		
Tumor thickness at TNI (mm)					
≤3	60	48.4	>51	0.023*	0.118
>3	64	51.6	19.4		
Fibrous capsule					
Present	43	34.4	27.8	0.468	–
Absent	81	65.6	25.8		
Perineural growth					
Present	11	8.9	50.2	0.539	–
Absent	113	91.1	27.5		
Vascular invasion					
Present	46	37.1	18.0	0.055	0.287
Absent	78	62.9	40.8		
Lymphatic invasion					
Present	33	26.6	19.4	0.280	–
Absent	91	73.4	29.2		
Bile duct invasion					
Present	11	8.8	27.8	0.624	–
Absent	113	91.2	27.5		

*DFS* disease-free survival, *CRLM* colorectal liver metastases, *UV* univariate, *MV* multivariate, *CEA* carcinoembryonic antigen, *DFI* disease-free interval between treatment of primary tumor and detection of the CRLM, *TNI* tumor–normal interface

* *p* < 0.05 was considered statistically significant

**Table 2:** Relation of clinical and histologic factors with OS after liver resection in patients with solitary CRLM

	*n*	%	Median OS	UV *p* value	MV *p* value
Size (mm)					
≤50	93	75	65.9	0.050*	0.004*
>50	31	25	29.2		
CEA (ng/ml)					
≤200	121	97.6	57.3	0.912	–
>200	3	2.4	28.9		
DFI					
≤12	72	58.1	49.0	0.059	0.019*
>12	52	41.9	91.5		
Nodal state primary					
N0	54	43.5	61.0	0.231	–
N+	70	56.5	44.6		
Adjuvant therapy					
No	106	85.5	57.2	0.955	–
Yes	18	14.5	29.2		
Tumor thickness at TNI (mm)					
≤3	60	48.4	95.3	0.043*	0.068
>3	64	51.6	48.8		
Fibrous capsule					
Present	43	34.4	109.3	0.037*	0.240
Absent	81	65.6	56.7		
Perineural growth					
Present	11	8.9	109.3	0.652	–
Absent	113	91.1	55.9		
Vascular invasion					
Present	46	37.1	48.8	0.483	–
Absent	78	62.9	58.2		
Lymphatic invasion					
Present	33	26.6	41.9	0.013*	0.041*
Absent	91	73.4	62.2		
Bile duct invasion					
Present	11	8.8	76.7	0.048*	0.094
Absent	113	91.2	55.9		

*OS* overall survival, *CRLM* colorectal liver metastases, *UV* univariate, *MV* multivariate, *CEA* carcinoembryonic antigen, *DFI* disease-free interval between treatment of primary tumor and detection of the CRLM, *TNI* tumor–normal interface

* *p* < 0.05 was considered statistically significant

Tumor thickness at tumor–normal interface varied between 0.1 and 7.2 mm, with a median of 3 mm, and was not correlated with the size of the liver metastases (*p* = 0.213). Although there was a significant association of increased thickness with decreased outcome (both DFS and OS) in univariate analysis, it was no longer significant in multivariate analysis (Tables 1, 2).

#### Intrahepatic Spread

Frequency of different forms of intrahepatic invasion varied; perineural growth (*n* = 11; 8.9 %) and bile duct invasion (*n* = 11; 8.8 %) were both relatively uncommon, whereas vascular and lymphatic invasion were seen more frequently (*n* = 46; 37.1 %, respectively *n* = 33; 26.6 %).

In univariate analysis, presence of bile duct invasion was associated with improved OS (76.7 vs. 55.9 months; *p* = 0.048), but this was not the case in multivariate analysis (*p* = 0.094). Presence of intrahepatic lymphatic invasion was correlated with a decreased median OS (41.9 vs. 62.2 months, *p* = 0.013), which remained significant in multivariate analysis (*p* = 0.041) (Supplementary Fig. 1a).

In the current study, no correlation between different forms of intrahepatic spread or between any of the histologic features and the various items of the clinical risk score was observed. However, there was a correlation between presence of a fibrous capsule and absence of intrahepatic vascular invasion (*p* = 0.014) and between presence of a fibrous capsule and presence of intrahepatic bile duct invasion (*p* = 0.013).

In 15 patients, a combination of intrahepatic lymphatic invasion and intrahepatic vascular invasion was present, and this combination was associated with a decreased OS (median 28.1 vs. 62.2 months) in univariate and multivariate analysis (*p* < 0.0001) (Supplementary Fig. 1b).

## Discussion

The current study describes the association between multiple histologic features in combination with clinical factors and survival in 124 patients who underwent liver resection for CRLM. A homogenous group of patients was evaluated because all patients underwent a complete resection (R0), for a solitary metastasis without neoadjuvant systemic treatment. The only significant histologic factor associated with decreased survival in multivariate analysis was presence of intrahepatic lymphatic invasion, especially in combination with intrahepatic vascular invasion.

Other authors also described lymphatic invasion as a negative predictor for survival.^13^,^18^,^20^ In the current study, we observed a relative high frequency of lymphatic invasion (26.6 %) compared to earlier studies (12–15 %).^18^,^20^ This might be due to the use of immunohistochemistry, which is supported by a recently published study with the same methodology and a similar frequency of lymphatic invasion (29 %).^18^,^20^,^34^–^36^ Presence of lymphatic invasion has been associated with spread to hepatic lymph nodes, which often leads to incurable disease.^20^,^37^ In the current study, the worse prognosis was demonstrated in patients with a combination of vascular and lymphatic invasion. This unfavorable combination has been observed before and might reflect a tumor with aggressive behavior.^23^

Another interesting finding from the current study was that the median tumor thickness at tumor–normal interface in patients who were not treated with neoadjuvant systemic therapy was 3.0 mm. This was only slightly higher than the tumor thickness of 2.8 mm described in patients treated with neoadjuvant chemotherapy.^26^ This raises the question whether tumor thickness at tumor–normal interface reflects chemotherapy response or tumor biology; this would be an interesting subject for further research.

A major strength of the present study is the inclusion of patients with solitary CRLM only, who were operated with complete margins (R0) to create an homogenous group of patients. Previous studies on histologic prognostic factors included patients with multiple CRLM and R1 resections as well, which might lead to significant bias of the results.^18^,^20^,^36^ First, heterogeneity of histologic features between the different liver metastases might exist and could lead to bias studying prognostic factors for survival. Second, patients who undergo R1 resection usually have a higher risk of local recurrences and have an impaired survival.^38^,^39^ Third, patients with multiple metastases have a significantly decreased survival, and number of metastases is the most important factor in the Fong classification for survival.^9^ By excluding these potential biases in the present study, the assessment of the prognostic histologic factors are more reliable.

Another strength is that this homogenous group of patients with solitary metastasis were not treated with neoadjuvant systemic therapy. In recent studies, patients with and without neoadjuvant systemic therapy were mixed, and conclusions were drawn from a population highly susceptible to bias.^25^,^36^,^40^ Neoadjuvant systemic therapy has a significant impact on tumor histology, and even prognostic factors such as resection margins might be less important.^27^,^28^,^41^ Because the detection of histologic prognostic factors in metastatic disease is still in its infancy and the effects of neoadjuvant systemic therapy on lymphatic invasion are unknown, a study with an homogeneous population should be a first step. However, there seems to be an increasing preference to utilize neoadjuvant systemic therapy for high risk patients, despite a lack of convincing evidence on survival benefit in patients with limited metastases.^42^–^44^ Therefore, a limitation of the present study is that the impact of lymphatic invasion on survival has to be confirmed in patients treated with neoadjuvant systemic therapy. In the total group of patients treated in our institution only 5 patients (3.8 %) with solitary metastasis were treated with neoadjuvant chemotherapy, which made it impossible to compare, but this should be the goal for future research.

In conclusion, intrahepatic lymphatic invasion, based on immunohistochemical detection of lymphatic vessels, is an adverse prognostic factor for OS in patients with a solitary CRLM. Therefore, we recommend evaluating the presence or absence of intrahepatic lymphatic and vascular invasion in the histologic assessment of CRLM. Future research is needed to determine whether adjuvant treatment strategies should be based on these adverse prognostic histologic factors.

## Electronic supplementary material

Supplementary material 1 (DOCX 171 kb)
