# Supplementary material for: Lymphatic Invasion is an Independent Adverse Prognostic Factor in Patients with Colorectal Liver Metastasis
Source: Ann Surg Oncol. 2015 May 19;22:638–45. doi: 10.1245/s10434-015-4562-8 (PMC4686554; doi:10.1245/s10434-015-4562-8)
Supplement: Supplementary file 1 [file 10434_2015_4562_MOESM1_ESM.docx]

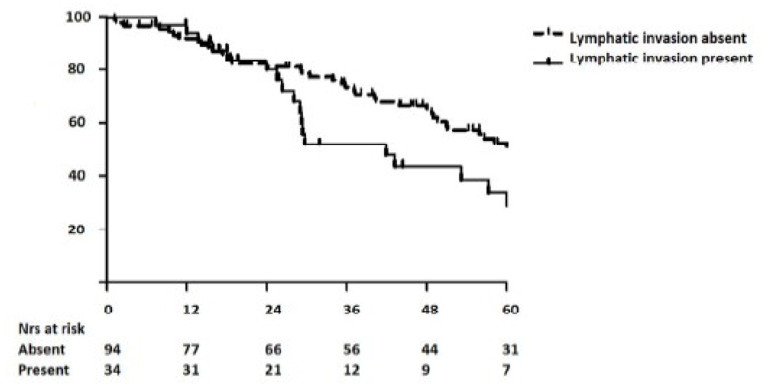

Supplemental figure 1a: Overall survival in months after liver resection in patients with and without intrahepatic lymphatic invasion (p=0.013). Survival in months on the X-axis and survival in percentage on the Y-axis.

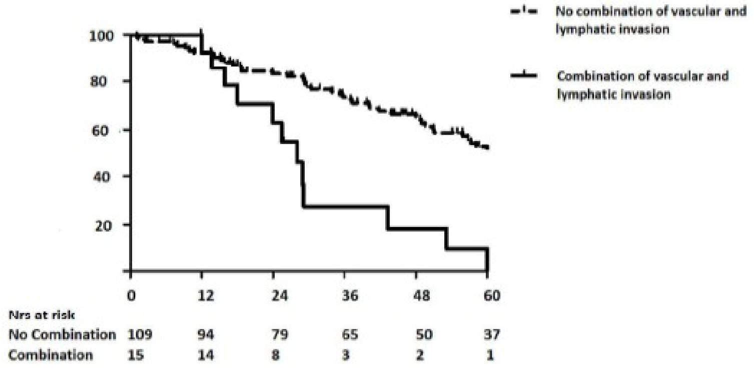

Supplemental figure 1b:
Overall survival in months after liver resection in patients with intrahepatic lymphatic invasion in combination with vascular invasion and in patients without this combination ($p<0.0001$). Overall survival in months on the X-axis and survival in percentage on the Y-axis.

Supplemental table 1

Antibody	Clone	Dilution	Ab-retrieval	Source
CD-31	M0823	1:40	EDTA	Dako, Denmark
D2-40	CM266C	1:50	EDTA	Biocare Medical, Belgium
CK-7	MU255-UC	1:800	Pronase	Klinipath, Netherlands
S-100	Z0311	1:2000	EDTA	Dako, Denmark
